# Plasma versus Erythrocyte Vitamin E in Renal Transplant Recipients, and Duality of Tocopherol Species

**DOI:** 10.3390/nu11112821

**Published:** 2019-11-19

**Authors:** Camilo G. Sotomayor, Ramón Rodrigo, António W. Gomes-Neto, Juan Guillermo Gormaz, Robert A. Pol, Isidor Minović, Manfred L. Eggersdorfer, Michel Vos, Ineke J. Riphagen, Martin H. de Borst, Ilja M. Nolte, Stefan P. Berger, Gerjan J. Navis, Stephan J. L. Bakker

**Affiliations:** 1 Division of Nephrology, Department of Internal Medicine, University Medical Center Groningen, University of Groningen, 9700 RB Groningen, The Netherlands; a.w.gomes.neto@umcg.nl (A.W.G.-N.); m.h.de.borst@umcg.nl (M.H.d.B.); s.p.berger@umcg.nl (S.P.B.); g.j.navis@umcg.nl (G.J.N.); s.j.l.bakker@umcg.nl (S.J.L.B.); 2 Molecular and Clinical Pharmacology Program, Institute of Biomedical Sciences, Faculty of Medicine, University of Chile, CP 8380453 Santiago, Chile; rrodrigo@med.uchile.cl; 3 Clínica Alemana de Santiago, Universidad del Desarrollo, 7610658 Santiago, Chile; jgormaz@gmail.com; 4 Division of Transplantation Surgery, University Medical Center Groningen, University of Groningen, 9700 RB Groningen, The Netherlands; r.pol@umcg.nl; 5 Department of Laboratory Medicine, University Medical Center Groningen, University of Groningen, 9700 RB Groningen, The Netherlands; i.minovic@umcg.nl (I.M.); m.l.eggersdorfer@umcg.nl (M.L.E.); m.j.vos01@umcg.nl (M.V.); i.j.riphagen@umcg.nl (I.J.R.); 6 Department of Epidemiology, University Medical Center Groningen, University of Groningen, 9700 RB Groningen, The Netherlands; i.m.nolte@umcg.nl

**Keywords:** vitamin E, α-tocopherol, γ-tocopherol, erythrocyte, inflammation, oxidative stress, cardiovascular disease, renal transplantation, renal transplant recipients

## Abstract

Redox imbalance is an adverse on-going phenomenon in renal transplant recipients (RTR). Vitamin E has important antioxidant properties that counterbalance its deleterious effects. However, plasma vitamin E affinity with lipids challenges interpretation of its levels. To test the hypothesis that erythrocyte membranes represent a lipids-independent specimen to estimate vitamin E status, we performed a cross-sectional study in a cohort of adult RTR (*n* = 113) recruited in a university setting (2015–2018). We compared crude and total lipids-standardized linear regression-derived coefficients of plasma and erythrocyte tocopherol species in relation to clinical and laboratory parameters. Strongly positive associations of fasting lipids with plasma tocopherol became inverse, rather than absent, in total lipids-standardized analyses, indicating potential overadjustment. Whilst, no variables from the lipids domain were associated with the tocopherol species measured from erythrocyte specimens. In relation to inflammatory status and clinical parameters with antioxidant activity, we found associations in directions that are consistent with either beneficial or adverse effects concerning α- or γ-tocopherol, respectively. In conclusion, erythrocytes offer a lipids-independent alternative to estimate vitamin E status and investigate its relationship with parameters over other biological domains. In RTR, α- and γ-tocopherol may serve as biomarkers of relatively lower or higher vulnerability to oxidative stress and inflammation, noticeably in opposite directions.

## 1. Introduction

Oxidative stress (OS) occurs when there is an imbalance between the generation of reactive oxygen species (ROS) and the antioxidant defense systems in the body, so that the latter becomes overwhelmed. It constitutes a unifying mechanism of injury of many types of disease processes, and it is causally involved in the pathogenesis of cardiovascular disease and malignancies by damaging biomolecules, such as lipids, proteins, and DNA. Of note, in stable renal transplant recipients (RTR), upon end-stage renal disease-related OS, elevated circulating metabolic by-products, maintenance of immunosuppressive therapy, chronic allograft rejection, new-onset diabetes after transplantation, and endothelial dysfunction are continuous and meaningful sources of ROS [1]. The role of free radicals and antioxidants in cardiovascular—particularly in atherogenesis—as well as in malignancy diseases is of encompassing relevance in the post-renal transplant setting, as both these conditions have been shown to currently dispute the leading individual causes of death in stable RTR [2,3,4], thus ultimately challenging efforts to improve long-term outcomes following renal transplantation [5]. 

Vitamin E is a lipid-soluble, chain-breaking type of antioxidant present in human blood. It has the potential to influence a broad range of mechanisms underlying human health and disease. Vitamin E works as a free radical scavenger and has the primary function of destroying peroxyl radicals. Thus, it protects long-chain polyunsaturated fatty acids (e.g., cell membranes or low-density lipoprotein cholesterol) from oxidation or destruction [6]. Its association with vitamin C is of great pathophysiological importance, because inhibition of lipid peroxidation by α-tocopherol occurs through its conversion into an oxidized α-tocopheroxyl radical, which in turn is regenerated to α-tocopherol through reduction by redox-active reagents, in particular vitamin C [7,8,9]. It is established that vitamin C and α-tocopherol interact as a network to protect lipids, proteins, and membranes from oxidative damage, wherein this interaction not only involves homogeneous solutions but also liposomal membrane systems, where vitamins C and E separately reside outside and within the membranes, respectively, with vitamin C acting as a synergist that reduces oxidized vitamin E and enhances its antioxidant activity, ultimately allowing for cooperative inhibition of oxidation [8,9]. This cooperative inhibition of oxidation is a protective mechanism of pathophysiological relevance that ultimately results in decreased formation of malondialdehyde [10]. 

α-tocopherol is the primary bioactive form of vitamin E, and is also best known for its role in human health. Seven other naturally occurring vitamin E compounds have been described: β-, γ-, and δ-tocopherol and α-, β-, γ-, and δ-tocotrienols. Reports on total tocopherol concentrations, which adjust for the bioavailability of the various forms, do not reflect the bioactivity of α-tocopherol, which is now used as the standard for dietary sufficiency [11]. Next, γ-tocopherol may have important adverse or beneficial effects; however, the overall health effects of γ-tocopherol have not been established.

One of the major problems hampering correct interpretation of the raw association between concentrations of vitamin E and high cardiovascular-risk conditions is attached to its lipoprotein-dependent circulating transportation. The affinity of vitamin E for circulating lipids results in a highly positive correlation with both fasting cholesterol and triglycerides concentrations [12,13]. The need to estimate vitamin E status has largely been addressed in clinical studies through performing analyses of the quotient of vitamin E over the sum of total cholesterol and fasting triglycerides, hereby negating the possible introduction of error from double correction for variance shared by total cholesterol and fasting triglycerides. The current study was conducted to test the hypothesis that erythrocyte vitamin E measurement may offer a lipids-independent specimen to estimate vitamin E status, and thus endeavor a more representative assessment of its associations with parameters over other biological domains. We aimed to compare coefficient estimates of the association of crude and total lipids-standardized plasma and erythrocyte vitamin E species with inflammatory status and clinical parameters with antioxidant activity in RTR, being a population of particularly high risk of cardiovascular and malignancy disease.

## 2. Materials and Methods 

### 2.1. Study Design and Population

For this study, we used preliminary data from the TransplantLines Prospective Cohort Study and Biobank of Solid Organ Transplant Recipients. In this ongoing study with a target follow-up duration of 30 years, from June 2015, all solid organ transplantation patients of the University Medical Center Groningen (UMCG, The Netherlands) were invited to participate. Exclusion criteria comprised no mastery of the Dutch language or cognitive dysfunction. Renal transplant recipients with complete plasma and erythrocyte vitamin E laboratory measurements were included in the analyses, resulting in 113 RTR, of whom the data is presented here. All patients provided signed written informed consent. The study protocol was approved by the Institutional Review Board (METc 2014/077), adheres to the UMCG Biobank Regulation, and is in accordance with the WMA Declaration of Helsinki and the Declaration of Istanbul. The cohort study is registered at clinicaltrials.gov (TransplantLines: The Transplantation Biobank, number NCT03272841). Full details on the rationale and study design are reported [14].

### 2.2. Data Collection, and Clinical and Laboratory Measurements

Medical history and medication use, including immunosuppressive therapy, were extracted from electronic hospital records. The measurement of clinical parameters has been described in detail [14]. Information about smoking status was collected from the self-administered Smoking Behaviour Questionnaire. Serum creatinine values were used to obtain the estimated glomerular filtration rate (eGFR) with the Chronic Kidney Disease Epidemiology Collaboration equation [15]. Blood was drawn in the morning after an 8 to 12-h overnight fasting period. Participants were instructed to collect a 24-h urine sample according to strict protocol on the day before their visit to the outpatient clinic; that is, discard their morning urine specimen, collect all subsequent urine throughout the next 24 h, and include the next morning’s first specimen of the day of the visit to the outpatient clinic. Blood drawing and receipt of the collected 24-h urine samples were performed by experienced nurses at our outpatient clinic. Serum levels of total cholesterol, high-density lipoprotein (HDL) cholesterol, low-density lipoprotein (LDL) cholesterol, and total triglycerides were quantified with validated routine enzymatic assays with spectrophotometric detection, all on a Roche Modular P chemistry analyzer (Roche, Basel, Switzerland). On the same analyzer, fasting blood glucose was measured via the hexokinase method. Serum uric acid and creatinine were measured via an enzymatic assay with colorimetric detection on a Roche Modular P chemistry analyzer (Roche, Basel, Switzerland). High-sensitivity C-reactive protein (hs-CRP) was determined by nephelometry (BN II system Siemens, Marburg, Germany). HbA_1C_ (glycated hemoglobin) was determined in ethylenediaminetetraacetic acid (EDTA)-anticoagulated whole blood. 

#### 2.2.1. Vitamin E in Plasma

Whole blood was collected in tubes containing Na_2_EDTA as anticoagulant and shielded from light. Plasma was collected after centrifugation and frozen at −20 °C until use. Analytes were extracted by supported liquid extraction, eluted in 5% isopropanol in heptane and evaporated under nitrogen flow, and resuspended in 250 µL of ethanol. Next, α-tocopherol and γ-tocopherol were separated by liquid chromatography using a Luna Phenyl-Hexyl column (Phenomenex, Utrecht, The Netherlands). Solvents used were 2 mM ammonium acetate dissolved in water with an 0.1% addition of formic acid and 10 mM ammonium acetate dissolved in ethanol/methanol mixture with a 0.1% addition of formic acid. Separation was followed by detection using a triple quad MS/MS system using deuterated internal standards [α-tocopherol-d6 (IsoSciences, Ambler, AK, USA) and γ-tocopherol-d4 (Toronto Research Chemicals Inc, North York, ON, Canada)].

#### 2.2.2. Vitamin E in Erythrocytes

Whole blood was collected in tubes containing Na_2_EDTA as anticoagulant and shielded from light. After centrifugation, erythrocytes were washed three times in PBS and resuspended to a haematocrit value of approximately 50%. In total, 500 µL of the suspension were mixed with an antioxidant mixture (0.4% pyrogallol, 0.05% butylated hydroxytoluene in methanol, and 2.5% ascorbic acid in EDTA buffer pH 5.4) and frozen at −20 °C until use. Erythrocyte cell counting was performed in the remaining suspension using a Sysmex XN-9000 module (Sysmex, Etten-Leur, The Netherlands). Samples were extracted using hexane, dried under nitrogen flow, and resuspended in 300 µL of ethanol. α-Tocopherol and γ-tocopherol were separated by liquid chromatography using a Symmetry C18 column (Waters Chromatography, Etten-Leur, The Netherlands) using a gradient of 0.01 M ammonium acetate, 40% water, 60% acetonitrile, 25% dichloromethane, 37.5% acetonitril, and 37.5% methanol. Separation was followed by detection at 292 nm using UV/VIS-spectroscopy using rac-Tocol (Abcam, Cambridge, UK) as an internal standard.

### 2.3. Statistical Analyses

All analyses were performed using IBM SPSS Statistics, version 23.0 for Windows software (IBM, Armonk, NY, USA). Data are expressed as mean ± standard deviation (SD) for normally distributed variables, and as median (IQR) for skewed variables. Categorical data are expressed as n (percentage). Age and sex-adjusted linear regression analyses were performed to examine the association of clinical and laboratory parameters with vitamin E species (i.e., α- and γ-tocopherol). Residuals were checked for normality and a natural log-transformation was applied when appropriate. For these analyses, crude and total lipids-standardized concentrations of vitamin E species measured in plasma and erythrocyte specimens were evaluated. Total lipids standardization was performed by calculating the quotient of vitamin E species over total lipids (i.e., sum of total cholesterol and triglycerides). All reported probability values are two-tailed, and a *p* ≤ 0.05 was considered statistically significant.

## 3. Results

### 3.1. Baseline Characteristics

**Figure 1:**
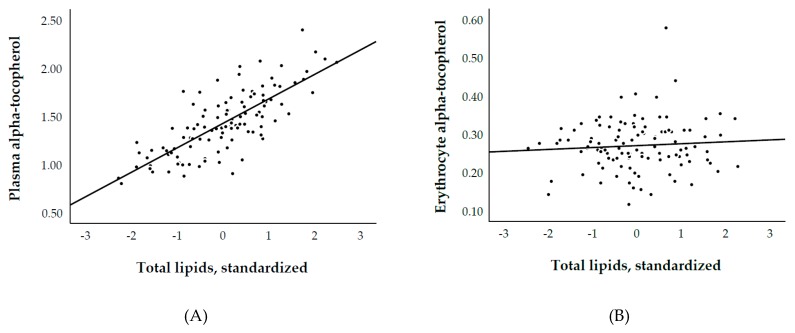
Association of (**A**) plasma (R^2^ = 0.60) and (**B**) erythrocyte (R^2^ = 0.01) α-tocopherol with total lipids. Plasma and erythrocyte α-tocopherol are expressed in mg/dL and mg/10^13^ erythrocytes, respectively.

**Figure 2:**
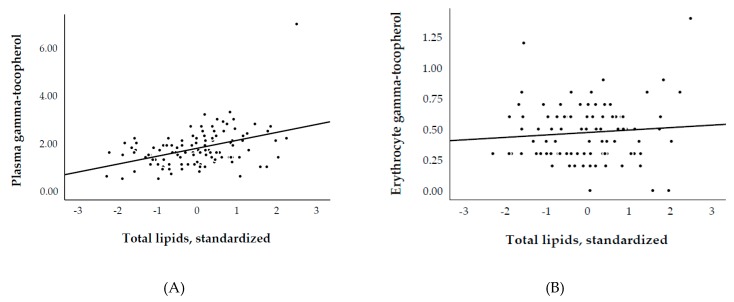
Association of (**A**) plasma (R^2^ = 0.18) and (**B**) erythrocyte (R^2^ = 0.01) γ-tocopherol with total lipids. Plasma and erythrocyte γ-tocopherol are expressed in mg/dL and mg/10^13^ erythrocytes, respectively.

We included 113 RTR with a median age of 55 ± 14 years, 60% males, and 76% Caucasian. Plasma and erythrocyte α-tocopherol concentrations were 1.4 ± 0.3 mg/dL and 0.27 ± 0.07 mg/10^13^ erythrocytes, respectively. Plasma α-tocopherol (mg/dL)/total lipids (g/dL) ratio was 4.2 ± 0.7. None of the study subjects were vitamin E deficient according to Horwitt et al. [16]. Plasma and erythrocyte γ-tocopherol concentrations were 0.07 ± 0.03 mg/dL and 0.02 ± 0.01 mg/10^13^ erythrocytes, respectively. We first investigated the associations of plasma and erythrocytes α- and γ-tocopherol with each other and with fasting lipids. We found positive associations between plasma α- and γ-tocopherol (std. β = 0.36; *p* < 0.001), as well as between erythrocytes α- and γ-tocopherol (std. β = 0.27; *p* = 0.01). Among different models used to study the association between α-tocopherol with fasting lipids as its potential independent determinants, we found that total lipids could explain 60% of the variation in plasma α-tocopherol, whereas it could explain <1% of the variation in erythrocyte α-tocopherol (Table 1, Model 4; Figure 1A,B). Following a similar pattern, although in a relatively smaller-scale, we found that total lipids could explain 18% of the variation in plasma γ-tocopherol, whereas it could explain <1% of the variation in erythrocyte γ-tocopherol (Table 2, Model 4, Figure 2A,B). Other combinations of lipids (Table 1 and Table 2; models 1–3, and 5–7) did not perform much better or worse than total lipids.

### 3.2. Plasma and Erythrocyte α-Tocopherol, and Biological Parameters

Linear regression analyses of age- and sex-adjusted associations between plasma and erythrocyte α-tocopherol and demographics, anthropometrics, allograft function, transplant-related, and laboratory parameters from the lipids, oxidative stress, and inflammatory biological domains are shown in Table 3. Otherwise, strongly positive associations of crude plasma α-tocopherol with fasting lipids were found to be significantly inverse, rather than absent, in total lipids-standardized plasma α-tocopherol analyses. On the other hand, no variables from the lipids domain were associated with crude erythrocyte α-tocopherol; whereas total lipids-standardized erythrocyte α-tocopherol was found to inversely and strongly associate with parameters over the lipids domain. Distinctive results were found upon analyses of the anti-oxidative agent high-density lipoprotein (HDL)-cholesterol. Whereas crude plasma α-tocopherol was not associated with HDL-cholesterol, total lipids-standardized plasma α-tocopherol, as well as crude erythrocyte α-tocopherol concentrations, were found to positively associate with HDL-cholesterol (Table 1, under the subheading *Antioxidants*, *pro-oxidants*, *and inflammation*). Although, both in plasma and erythrocyte specimens, we found an inverse association between uric and total-lipids standardized α-tocopherol, this finding did not remain over non-standardized analyses of erythrocyte α-tocopherol. Noteworthy, the anti-inflammatory and redox-active agent vitamin C directly associated with total lipids-standardized plasma and erythrocyte α-tocopherol. This association was consistent over non-standardized analyses of erythrocyte α-tocopherol. Finally, either in plasma or erythrocyte specimens, an inverse association between glucose and α-tocopherol was found, but this association was not consistent in non-standardized analyses of erythrocyte α-tocopherol, indicating potential overadjustment.

### 3.3. Plasma and Erythrocyte γ-Tocopherol, and Biological Parameters

Linear regression analyses of age- and sex-adjusted associations between plasma and erythrocyte γ-tocopherol and demographics, anthropometrics, allograft function, transplant-related, and laboratory parameters from the lipids, oxidative stress, and inflammatory biological domains are shown in Table 4. Analogous to α-tocopherol analyses, otherwise, strongly positive associations of crude plasma γ-tocopherol with fasting lipids were found to be significantly inverse, rather than absent, in total lipids-standardized plasma and erythrocyte γ-tocopherol analyses, whereas no significant associations were observed in crude analyses of the γ-tocopherol concentrations measured from erythrocyte specimens. Noteworthy, the inflammatory biomarker hs-CRP was directly associated with total lipids-standardized plasma and erythrocyte γ-tocopherol. This association was consistent over non-standardized analyses of erythrocyte γ-tocopherol.

## 4. Discussion

In the current cohort study of RTR, we found that total lipids (i.e., total cholesterol plus triglycerides) significantly explained 60% and 18% of the variation in plasma α- and γ-tocopherol, whereas they only explained <1% of the variation in α- and γ-tocopherol measured from erythrocryte specimens. As for the association of plasma α- and γ-tocopherol species with fasting lipids, we consistently found an inversion of standardized regression coefficients from positive to negative values in lipids-standardized analyses, indicating potential overadjustment. In turn, otherwise, absent associations of erythrocyte α- and γ-tocopherol concentrations with parameters over the lipids domain consistently became inverse in total lipids-standardized analyses, which may likewise be indicative of overcorrection. These findings leave the conclusion that vitamin E species measured from erythrocytes may offer a lipids-independent specimen to estimate vitamin E status, and thus endeavor a more representative assessment to investigate its association with parameters over other biological domains.

Within the lipids domain, HDL cholesterol contributes to total cholesterol and thus it may likely be expected to positively correlate with total cholesterol. However, because of the action of circulating cholesterol ester transfer protein, it may be anticipated to inversely correlate with fasting triglycerides [17]. Moreover, because of its antioxidant activity [18,19,20], one would foresee a positive association of HDL cholesterol with vitamin E. Indeed, in agreement with the rather outlier pattern expected for HDL cholesterol in comparison to other lipid parameters, we found it to positively associate with total lipids-standardized plasma α-tocopherol, with a consistent pattern over non-standardized analyses with erythrocyte α-tocopherol. Interestingly, this pattern is similar to that observed for the radical scavenger type of antioxidant, vitamin C. These findings are particularly relevant in the post-kidney transplantation setting, as we have previously shown that both HDL cholesterol efflux and vitamin C status are associated with long-term kidney graft and patient survival, respectively [21,22]. 

We found the associations between α-tocopherol and γ-tocopherol concentrations to be positive, in both analyses specimens. Whilst, interestingly, the physiological regulatory direction of associations of γ-tocopherol with biological variables, if present, were opposite to those observed for α-tocopherol. α-tocopherol directly associated with HDL-cholesterol and vitamin C, and inversely with uric acid. On the other hand, irrespective of standardization methods, γ-tocopherol directly associated with hs-CRP. Previous studies have reported contrapositive associations of α- and γ-tocopherol with parameters over different biological domains, particularly in relation to inflammation, oxidative stress, and aging [23,24,25,26,27,28,29]. In agreement with our findings, these studies reported results in directions that are consistent with beneficial effects concerning α-tocopherol, and adverse outcomes concerning γ-tocopherol. Specific intracellular mechanisms for either anti- or pro-inflammatory functions of these tocopherols species have been proposed by Cook-Mills et al. [30,31,32]. While recruitment of lymphocytes and eosinophils is inhibited by α-tocopherol, it has been shown that γ-tocopherol elevates migration of leukocytes through intercellular adhesion molecule-1 (ICAM-1) and vascular cell adhesion molecule-1 (VCAM-1)-dependent mechanisms at the endothelium level. Upon direct binding of protein kinase Cα (PKCα), α- and γ-tocopherol, furthermore, have been shown to differentially regulate downstream intracellular signaling of these molecules, which is the proposed mechanistic background to explain the demonstrated contrapositive regulatory biological directions of these vitamin E isoforms [30,31,32]. Remarkably, these opposing regulatory functions of vitamin E isoforms may have considerable impact on the interpretation of a broad number of clinical studies on vitamin E performed over the last decades, which remains to be reviewed and critically analyzed. 

The complexity of the antioxidants and pro-oxidants network is partly underscored by the action of one antioxidant depending on the proper function of other members of the antioxidant system, and to a considerable extent also articulated by physicochemical conditions of microenvironments in biological fluids. Uric acid, despite being an endogenous antioxidant that accounts for about 70% of the total antioxidant capacity of plasma [33], has largely been shown to associate and predict development of hypertension, atherosclerosis, visceral obesity, insulin resistance, dyslipidemia, diabetes type II, kidney disease, and cardiovascular events [34,35,36,37,38,39,40,41]. This paradox may rise from the duality of activities displayed by uric acid in either hydrophilic or hydrophobic physicochemical microenvironments. Considerations of uric acid regarding analyses at different compartments and at different levels of biological organization point towards a contributory role of its pro-oxidative actions in common pathogenic pathways leading to cardiovascular disease in chronic kidney disease patients. Thus, the inverse association of plasma and erythrocytes α-tocopherol with uric acid in total-lipids standardized analyses, which was not consistent over non-standardized analyses in erythrocytes, seems to be in line with the proposed protective effects of α-tocopherol against potentially deleterious pro-oxidant activity of uric acid, whilst they underline the relevance of accounting for lipophilic conditions in the study of uric acid in relation to α-tocopherol and within the complex antioxidants network.

To our knowledge, the current study is the first study to investigate circulating levels of α- and γ-tocopherol species measured in plasma, as well as in erythrocyte specimens. The latter may allow a total lipids-independent vitamin E status estimation to investigate its relation to inflammatory status and clinical parameters with antioxidant activity in RTR. This is relevant because RTR are a population at particular vulnerability to oxidative damage on the basis that cardiovascular and malignancy disease are major hazards in the post-renal transplantation setting [2,3,4,5]. It should be acknowledged that we did not have access to data on urinary metabolites of vitamin E species, which could have given us further information on system vitamin E status [42]. Next, our study is cross-sectional in nature, which precludes us from drawing hard conclusions about cause-and-effect relations. Whereas we evaluated routinely measured clinical parameters with established antioxidant activity, the association with markers of oxidative damage remains to be further studied. Upon these preliminary results, nonetheless, forthcoming analyses of the data to be generated by the long-lasting TransplantLines Prospective Cohort Study and Biobank of Solid Organ Transplant Recipients [14] are warranted, in order to longitudinally relate vitamin E status, evaluated by means of α- and γ-tocopherol measured in erythrocytes membranes, to inflammatory state and oxidative stress by means of total antioxidant capacity of plasma and lipid peroxidation biomarkers, and to assess its association to hard long-term outcomes (e.g., overall and cause-specific graft and recipient loss), and to be able to likewise account for liver, lung, and heart transplant recipients.

## 5. Conclusions

In conclusion, erythrocytes offer a lipids-independent alternative to estimate vitamin E status and investigate its relationship with parameters over other biological domains. Despite a positive association between α- and γ-tocopherol themselves in both analyzed specimens (i.e., plasma and erythrocytes), we found intriguing opposing associations of α- and γ-tocopherol in the biological domains of inflammation and redox imbalance. The current findings support the notion that, in opposite regulatory biological directions, α- and γ-tocopherol, respectively, have the characteristics of biomarkers associated with a relatively lower and higher vulnerability to these long-term ongoing pathogenic phenomena in RTR. Further studies are warranted to evaluate whether considerate supplementation of vitamin E species may represent a not yet appropriately recognized opportunity to decrease the burden by long-term low-grade inflammation and persistent oxidative challenge, and its deleterious consequences in RTR.

## Figures and Tables

**Table 1:** Association between plasma and erythrocytes α-tocopherol and fasting lipids in renal transplant recipients (*n* = 113).

**Plasma α-tocopherol**	**Model 1**	**Model 2**	**Model 3**	**Model 4**	**Model 5**	**Model 6**	**Model 7**
Total cholesterol	0.68 ***	—	0.53 ***	—	0.49 ***	—	—
HDL cholesterol	—	—	—	—	0.10	0.29 ***	0.32 ***
Non-HDL cholesterol	—	—	—	—	—	—	0.49 ***
LDL cholesterol	—	—	—	—	—	0.41 ***	—
Triglycerides	—	0.61 ***	0.43 ***	—	0.48 ***	0.59 ***	0.48 ***
Total lipids	—	—	—	0.77 ***	—	—	—
*R^2^*	0.46	0.37	0.62	0.60	0.63	0.60	0.63
**Erythrocytes α-tocopherol**	**Model 1**	**Model 2**	**Model 3**	**Model 4**	**Model 5**	**Model 6**	**Model 7**
Total cholesterol	0.08	—	0.15	—	0.06	—	—
HDL cholesterol	—	—	—	—	0.21	0.24 *	0.24 *
Non-HDL cholesterol	—	—	—	—	—	—	0.06
LDL cholesterol	—	—	—	—	—	0.05	—
Triglycerides	—	−0.17	−0.22 *	—	−0.13	−0.11	−0.13
Total lipids	—	—	—	−0.08	—	—	—
*R^2^*	0.01	0.03	0.05	0.01	0.08	0.08	0.08

Associations between α-tocopherol and lipids were tested via univariable and multivariable linear regression analyses of which standardized β coefficients are presented (* *p* < 0.05, *** *p* < 0.001).

**Table 2:** Association between plasma and erythrocytes γ-tocopherol and fasting lipids in renal transplant recipients (*n* = 113).

**Plasma γ-tocopherol**	**Model 1**	**Model 2**	**Model 3**	**Model 4**	**Model 5**	**Model 6**	**Model 7**
Total cholesterol	0.20 *	—	0.08	—	0.10	—	—
HDL cholesterol	—	—	—	—	–0.05	–0.01	<0.001
Non-HDL cholesterol	—	—	—	—	—	—	0.10
LDL cholesterol	—	—	—	—	—	0.05	—
Triglycerides	—	0.39	0.37 ***	—	0.35 **	0.38 ***	0.35 **
Total lipids	—	—	—	0.42 ***	—	—	—
*R^2^*	0.04	0.16	0.16	0.18	0.16	0.16	0.16
**Erythrocytes γ-tocopherol**	**Model 1**	**Model 2**	**Model 3**	**Model 4**	**Model 5**	**Model 6**	**Model 7**
Total cholesterol	0.06	—	0.05	—	0.09	—	—
HDL cholesterol	—	—	—	—	–0.10	–0.07	–0.06
Non-HDL cholesterol	—	—	—	—	—	—	0.09
LDL cholesterol	—	—	—	—	—	0.08	—
Triglycerides	—	0.05	0.04	—	−0.01	0.01	–0.01
Total lipids	—	—	—	0.09	—	—	—
*R^2^*	0.004	0.003	0.01	0.01	0.01	0.01	0.01

Associations between γ-tocopherol and lipids were tested via univariable and multivariable linear regression analyses of which standardized β coefficients are presented (* *p* < 0.05, ** *p* < 0.01, *** *p* < 0.001).

**Table 3:** Baseline characteristics of renal transplant recipients, and their associations with non-standardized and total lipids-standardized plasma and erythrocyte α-tocopherol concentrations.

Baseline Characteristics	Overall RTR (*n* = 113)	α-tocopherol			
		Plasma		Erythrocyte	
		Standardization		Standardization	
		None	Lipids	None	Lipids
Plasma α-tocopherol, mg/dL, mean (SD)	1.4(0.3)	—	—	—	—
Quotient plasma α-tocopherol (mg/dL)/total lipids (g/dL), mean (SD)	4.2(0.7)	—	—	—	—
Erythrocyte α-tocopherol, mg/10^13^ erythrocytes, mean (SD) ^a^	0.27(0.07)	—	—	—	—
Quotient erythrocyte α-tocopherol (mg/10^13^ erythrocytes)/total lipids (g/dL), median (IQR) ^a^	0.79(0.61–1.04)	—	—	—	—
**Demographics and anthropometrics**					
Age, years, mean (SD) ^†^	55(14)	0.20 *	0.20 *	0.12	0.05
Gender, male, *n* (%) ^†^	68(60)	0.15	0.19 *	0.13	0.08
Ethnicity, Caucasian, *n* (%) ^b^	86(76)	−0.13	0.13	0.10	0.19
Body mass index, kg/m^2^, median (IQR) ^c^	25.9(24.2–29.3)	0.04	−0.17	−0.10	−0.17
Systolic blood pressure, mmHg, mean (SD) ^c^	140(19)	0.06	0.04	0.003	−0.01
Diastolic blood pressure, mmHg, mean (SD) ^c^	81(15)	0.002	−0.06	−0.17	−0.13
Diabetes mellitus, *n* (%) ^d^	31(27)	0.01	−0.04	0.08	−0.01
Current smoker, *n* (%) ^e^	5(4)	−0.04	0.01	−0.01	0.02
**Allograft function and transplantation**					
Creatinine, mg/dL, mean (SD)	1.5(0.4)	0.09	−0.04	0.01	−0.05
eGFR, mL/min/1.73 m^2^, mean (SD)	51(16)	−0.11	0.001	−0.08	0.01
Dialysis vintage ^d^					
*<1 year, n (%)*	62(56)	—	—	—	—
*1–5 years, n (%)*	37(33)	−0.13	−0.13	−0.16	−0.06
*>5 years, n (%)*	11(10)	0.17	0.06	0.01	−0.06
Time since transplantation, years, median (IQR) ^f^	1(1–10)	0.20*	0.07	0.02	−0.08
**Lipids**					
Total cholesterol, mg/dL, mean (SD)	181(38)	0.66 ***	−0.14	0.06	−0.46 ***
Non-HDL cholesterol, mg/dL, mean (SD)	127(37)	0.67 ***	−0.24 **	−0.03	−0.54 ***
LDL cholesterol, mg/dL, mean (SD)	110(35)	0.57 ***	−0.13	0.01	−0.41 ***
Triglycerides, mg/dL, median (IQR)	151(101–197)	0.61 ***	−0.59 ***	−0.17	−0.74 ***
Total lipid, mg/dL, median (IQR)	334(272–397)	0.76 ***	−0.51 ***	−0.08	−0.76 ***
**Antioxidants, pro-oxidants, and inflammation**					
HDL cholesterol, mg/dL, median (IQR)	50(41–64)	−0.01	0.25 *	0.24*	0.21
γ-Glutamyltransferase, U/L, median (IQR)	30(19–42)	0.05	−0.05	−0.06	−0.10
Uric acid, mg/dL, mean (SD)	6.4(1.6)	0.20	−0.26 **	−0.18	−0.35 ***
Vitamin C, mg/dL, median (IQR) ^g^	0.7(0.4–0.9)	0.10	0.22 *	0.23*	0.23 *
hs-CRP, mg/L, median (IQR) ^h^	3.0(1.3–7.0)	−0.11	0.09	−0.08	0.06
**Glucose homeostasis**					
Glucose, mg/dL, median (IQR)	99(90–114)	0.13	−0.21 *	−0.04	−0.31 *
HbA_1C_, %, median (IQR)	5.8(5.4–6.5)	0.09	0.09	0.02	−0.03

Associations between baseline characteristics and plasma and erythrocyte α-tocopherol concentration were tested via multivariable age- and sex-adjusted linear regression analyses, of which standardized β coefficients are presented (* *p* < 0.05, ** *p* < 0.01, and *** *p* < 0.001). Data available in ^a^ 111, ^b^ 90, ^c^ 112, ^d^ 110, ^e^ 55, ^f^ 109, ^g^ 99, and ^h^ 105 patients. ^†^ Associations were adjusted for age or gender, where applicable. Abbreviations: hs-CRP, high-sensitivity C-reactive protein; eGFR, estimated glomerular filtration rate.

**Table 4:** Baseline characteristics of renal transplant recipients, and their associations with non-standardized and total lipids-standardized plasma and erythrocyte γ-tocopherol concentrations.

Baseline Characteristics	Overall RTR (*n* = 113)	γ-tocopherol			
		Plasma		Erythrocyte	
		Standardization		Standardization	
		None	Lipids	None	Lipids
Plasma γ-tocopherol, mg/dL, mean (SD) ^a^	0.07(0.03)	—	—	—	—
Quotient plasma γ-tocopherol (mg/dL)/total lipids (g/dL), mean (SD) ^a^	0.22(0.08)	—	—	—	—
Erythrocyte α-tocopherol, mg/10^13^ erythrocyte, median (IQR) ^b^	0.02(0.01)	—	—	—	—
Quotient erythrocyte α-tocopherol (mg/10^13^ erythrocyte)/total lipids (g/dL), median (IQR) ^b^	0.06(0.03)	—	—	—	—
**Demographics and anthropometrics**					
Age, years, mean (SD) ^†^	55(14)	−0.10	−0.09	−0.14	−0.16
Gender, male, *n* (%) ^†^	68(60)	0.13	0.10	0.08	0.06
Ethnicity, Caucasian, *n* (%) ^c^	86(76)	−0.03	0.16	0.16	0.27
Body mass index, kg/m^2^, median (IQR) ^a^	25.9(24.2–29.3)	0.29 **	0.16	0.07	−0.07
Systolic blood pressure, mmHg, mean (SD) ^a^	140(19)	−0.09	−0.11	−0.12	−0.12
Diastolic blood pressure, mmHg, mean (SD) ^a^	81(15)	0.11	0.03	−0.09	−0.14
Diabetes mellitus, *n* (%) ^d^	31(27)	0.17	0.09	0.11	0.04
Current smoker, *n* (%) ^e^	5(4)	0.05	0.13	0.10	0.09
**Allograft function and transplantation**					
Creatinine, mg/dL, mean (SD)	1.5(0.4)	–0.05	–0.17	–0.05	–0.40
eGFR, mL/min/1.73 m^2^, mean (SD)	51(16)	0.07	0.18	0.03	0.08
Dialysis vintage ^d^					
*<1 year, n (%)*	62(56)	—	—	—	—
*1–5 years, n (%)*	37(33)	0.10	0.12	0.002	0.03
*>5 years, n (%)*	11(10)	−0.10	−0.15	−0.02	−0.07
Time since transplantation, years, median (IQR) ^b^	1(1–10)	0.08	−0.02	0.02	−0.04
**Lipids**					
Total cholesterol, mg/dL, mean (SD)	181(38)	0.19 *	−0.23 *	0.07	−0.29 **
Non-HDL cholesterol, mg/dL, mean (SD)	127(37)	0.28 **	−0.21 *	0.12	−0.29 **
LDL cholesterol, mg/dL, mean (SD)	110(35)	0.16	−0.21 *	0.10	−0.21 *
Triglycerides, mg/dL, median (IQR)	151(101–197)	0.40 ***	−0.22 *	0.06	−0.44 ***
Total lipid, mg/dL, median (IQR)	334(272–397)	0.42 ***	−0.2 5**	0.10	−0.45 ***
**Antioxidants, pro-oxidants, and inflammation**					
HDL cholesterol, mg/dL, median (IQR)	50(41–64)	−0.20 *	−0.07	−0.12	−0.02
γ-Glutamyltransferase, U/L, median (IQR)	30(19–42)	0.05	−0.003	−0.03	−0.03
Uric acid, mg/dL, mean (SD)	6.4(1.6)	0.10	−0.16	−0.04	−0.18
Vitamin C, mg/dL, median (IQR) ^f^	0.7(0.4–0.9)	−0.17	−0.05	0.03	0.12
hs-CRP, mg/L, median (IQR) ^g^	3.0(1.3–7.0)	0.14	0.23 *	0.24 *	0.20 *
**Glucose homeostasis**					
Glucose, mg/dL, median (IQR)	99(90–114)	0.21 *	0.01	0.13	−0.05
HbA_1C_, %, median (IQR)	5.8(5.4–6.5)	0.11	0.07	−0.001	−0.04

Associations between baseline characteristics and plasma and erythrocyte γ-tocopherol concentration were tested via multivariable age- and sex-adjusted linear regression analyses, of which standardized β coefficients are presented (* *p* <0.05, ** *p* <0.01, and *** *p* <0.001). Data available in ^a^ 112, ^b^ 109, ^c^ 90, ^d^ 110, ^e^ 55, ^f^ 99 and ^g^ 105 patients. ^†^ Associations were adjusted for age or gender, where applicable. Abbreviations: hs-CRP, high-sensitivity C-reactive protein; eGFR, estimated glomerular filtration rate.
